# Chordoid glioma of the third ventricle

**DOI:** 10.1590/0100-3984.2014.0125

**Published:** 2015

**Authors:** Marília Henrique Destefani, Alessandro Spanó Mello, Ricardo Santos de Oliveira, Gustavo Novelino Simão

**Affiliations:** 1 Cedirp – Radiologia e Diagnóstico por Imagem, Ribeirão Preto, SP, Brazil.; 2 Hospital das Clínicas – Faculdade de Medicina de Ribeirão Preto da Universidade de São Paulo (HCFMRP-USP), and Cedirp – Radiologia e Diagnóstico por Imagem, Ribeirão Preto, SP, Brazil.; 3 Hospital das Clínicas – Faculdade de Medicina de Ribeirão Preto da Universidade de São Paulo (HCFMRP-USP), Ribeirão Preto, SP, Brazil.

*Dear Editor*,

A previously healthy 27-year-old man was referred with an 8-month history of headaches, memory loss, progressive weight gain (obesity), hyperphagia and behavior changes.

Computed tomography (CT) scans revealed the presence of a midline, solid, and homogeneously enhancing mass involving the anterior aspect of the third ventricle.

**Figure 1:**
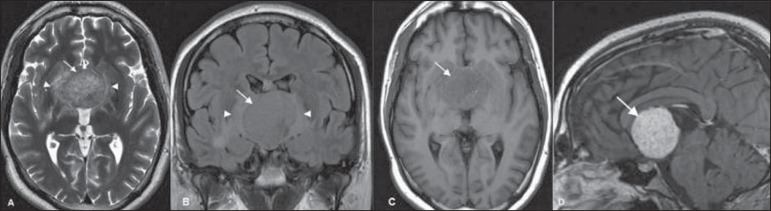
Axial MRI T2-weighted (**A**) and coronal FLAIR (**B**) sequences reveal a slightly hyperintense, well-defined hypothalamic/third ventricular tumor (arrows), with perilesional vasogenic edema (arrowheads). **C:** Axial MRI T1-weighted sequence reveals a predominantly isointense tumor (arrow). **D:** Gadolinium-enhanced sagittal MRI T1-weighted sequence reveals the tumor with uniform contrast enhancement (arrow).

Brain magnetic resonance imaging (MRI) (Figure 1) showed a well-defined, rounded mass in the third ventricle, measuring about 4.0 cm in the craniocaudal axis. The tumor was slightly heterogeneous, predominantly isointense at T1- and T2-weighted MRI sequences, presenting with diffuse enhancement after gadolinium injection. Perilesional vasogenic edema, compression and subsequent displacement of midbrain and hypothalamic structures were observed.

A subtotal resection of the tumor was microsurgically performed by interhemispheric transcallosal approach to the third ventricle.

The tumor was histologically classified as a chordoid glioma. The mass showed nests of regular epithelioid cells with large nuclei, prominent nucleoli, and abundant eosinophilic cytoplasm, within a myxoid stroma. Sparse lymphocytic infiltrate was present. Immunohistochemical studies demonstrated diffuse cytoplasmic expression for glial fibrillary acidic protein, vimentin, and CD34.

The patient died three months after surgery as a consequence of massive hypothalamic invasion combined with pneumonia.

Chordoid glioma is an unusual, noninvasive and slow-growing tumor that arises from the anterior third ventricle, frequently adherent to the hypothalamus^(1)^. There are reports in the literature about chordoid gliomas in other locations, such as the temporoparietal region, left thalamus and the corona radiata/thalamus^(2,3)^, most of them affecting children^(2)^.

It is typically a well-circumscribed, round or oval-shaped tumor, with greatest diameter in the craniocaudal direction. The tumor is hyperdense to the gray matter at CT, isointense at MRI T1-weighted sequences, and isointense to slightly hyperintense at MRI long-TR, with strong, uniform enhancement after contrast agent administration^(1,2,4-6)^. Cystic changes and necrosis may be present^(2,5,7)^. Calcifications are usually rare^(2,5,7)^. Usually, bilateral and symmetric perilesional vasogenic edema may also be observed^(3-5)^.

Given the tumor location, patients usually present with signs and symptoms related to obstructive hydrocephalus, such as nausea and headache, although endocrine imbalance, visual disturbances, behavior disorders and autonomic dysfunction are also reported in the literature^(1,4-6)^.

The histological and immunohistochemical features of these tumors are very typical and uniform, characterized by cords of oval to polygonal epithelioid cells with abundant eosinophilic cytoplasm and avid staining for glial fibrillary acidic protein and vimentin^(1,2,4)^.

The differential diagnosis includes masses of suprasellar region, such as pituitary macroadenoma, craniopharyngioma, optic and hypothalamic pilocytic astrocytoma, meningioma, ependymoma and lymphoma^(2,4)^.

Currently, the treatment of choice is complete surgical resection of the tumor^(1,4,6)^. Adjuvant radiotherapy has been used following subtotal resection^(2)^.

Despite being a low-grade tumor, the prognosis is usually poor because of its location and the difficulty in obtaining complete surgical resection without causing severe hypothalamic symptoms^(4)^. On the other hand, partial resection of the tumor is associated with high recurrence rates^(4-6)^.
